# Delays in diagnosis of young females with symptomatic cervical cancer in England: an interview-based study

**DOI:** 10.3399/bjgp14X681757

**Published:** 2014-09-29

**Authors:** Anita W Lim, Amanda J Ramirez, William Hamilton, Peter Sasieni, Julietta Patnick, Lindsay JL Forbes

**Affiliations:** Kings College London Promoting Early Cancer Presentation Group, King’s College London, London.; Kings College London Promoting Early Cancer Presentation Group, King’s College London, London.; University of Exeter Medical School, Primary care diagnostics, Exeter.; Centre for Cancer Prevention, Wolfson Institute of Preventive Medicine, Queen Mary University of London, Barts & The London School of Medicine and Dentistry, London.; Public Health England, NHS Cancer Screening Programmes, Sheffield.; Kings College London Promoting Early Cancer Presentation Group, King’s College London, London.

**Keywords:** cervical cancer, delays, early diagnosis, symptoms, young females

## Abstract

**Background:**

Diagnosis may be delayed in young females with cervical cancer because of a failure to recognise symptoms.

**Aim:**

To examine the extent and determinants of delays in diagnosis of young females with symptomatic cervical cancer.

**Design and setting:**

A national descriptive study of time from symptoms to diagnosis of cervical cancer and risk factors for delay in diagnosis at all hospitals diagnosing cervical cancer in England.

**Method:**

One-hundred and twenty-eight patients <30 years with a recent diagnosis of cervical cancer were interviewed. Patient delay was defined as ≥3 months from symptom onset to first presentation and provider delay as ≥ 3 months from first presentation to diagnosis.

**Results:**

Forty (31%) patients had presented symptomatically: 11 (28%) delayed presentation. Patient delay was more common in patients <25 than patients aged 25–29 (40% versus 15%, *P* = 0.16). Vaginal discharge was more common among patients who delayed presentation than those who did not; many reported not recognising this as a possible cancer symptom. Provider delay was reported by 24/40 (60%); in some no report was found in primary care records of a visual inspection of the cervix and some did not re-attend after the first presentation for several months. Gynaecological symptoms were common (84%) among patients who presented via screening.

**Conclusions:**

Young females with cervical cancer frequently delay presentation, and not recognising symptoms as serious may increase the risk of delay. Delay in diagnosis after first presentation is also common. There is some evidence that UK guidelines for managing young females with abnormal bleeding are not being followed.

## INTRODUCTION

The NHS in England offers routine cervical screening to females aged 25–64 years. Screening starts at the age of 25 years, based on evidence that harms outweigh benefits in females <25 years.^1^ The policy on the timing of the first invitation has been controversial, with many calling for females to be invited at a younger age. Females in Scotland (and, until recently, in Wales) are still invited from age 20 years. In 2009 the policy was reviewed, and retained, after public and professional debate around the time of the death of the celebrity Jade Goody from cervical cancer at the age of 27.

After this debate, the English Advisory Committee on Cervical Screening recommended that the routes to diagnosis and duration of symptoms in young patients with symptomatic cervical cancer be audited. This was based on case reports from clinicians and patients^2^,^3^ that delayed diagnosis resulted from failure (among patients and health professionals) to recognise the seriousness of symptoms, rather than from not being screened.^4^

The most common symptoms of cervical cancer are postcoital bleeding, intermenstrual bleeding, and vaginal discharge;^5^–^9^ however, these are common in young females with non-malignant conditions (for example, genital infections)^6^,^10^,^11^ or taking hormonal contraceptives. Furthermore, cervical cancer is very rare in young females: 353 females aged 25–29 years and 47 aged 20–24 years were diagnosed in 2011 in England.^12^

This study aimed to collect data on nature and duration of symptoms and risk factors for delay in presentation and diagnosis in young females with cervical cancer. This was intended to inform approaches to promote early presentation and prompt referral among young females with symptoms of cervical cancer.

## METHOD

### Study participants

The participants were females aged 18–29 years and diagnosed in England with cervical cancer during 2011 and 2012. Patients aged 25–29 years were included because many are diagnosed via symptomatic presentation^13^ (screening coverage is low (∼60%).^14^ Patients were excluded if they required an interpreter, or were inappropriate to interview (for example, if they had mental health issues).

Between April 2011 and March 2012, patients were prospectively identified by asking pathology laboratories and gynaeoncology clinical teams in all NHS hospitals diagnosing cervical cancer in England to notify the study investigators of eligible cases. The study was also publicised using cancer charity websites, so that patients could volunteer to take part directly. Diagnosis was confirmed for all patients by the pathology laboratory or clinical team. Each patient’s clinical team was asked to assess eligibility and invite her to take part. For patients providing specific consent, primary care records were requested for the 2 years before diagnosis, plus results of cervical screening tests from the National Cervical Screening Database.

**How this fits in**

There have been reports that young females with cervical cancer experience delays in diagnosis because of a failure to recognise symptoms. This study has found that a high proportion of patients aged <30 years with symptomatic cervical cancer delay presentation. Delay in referral after presentation is also common. There is some evidence that the UK guidelines for managing young females with abnormal bleeding are not being followed.

### Measures

Data were collected using a semi-structured interview-based measure (the interview schedule is available from the authors on request).^15^ This approach has advantages over collecting data from medical records or using self-completion questionnaires, because it allows the interviewer to clarify and check the sequence of events, and to probe for information that patients might not consider relevant.^16^

The measure collects data about dates of symptoms and healthcare attendances from the first possible cancer symptom through to diagnosis and risk factors for delay in presentation.

### Data collection

The same trained interviewer conducted all the interviews either face-to-face or on the telephone (according to the patients’ preference) within 2–6 months of diagnosis. Interviews were audiorecorded for quality assurance and data checking.

### Analysis

Characteristics (age, stage [using the International Federation of Gynecology and Obstetrics {FIGO} classification], histology, and geographical region) of patients who were interviewed were compared with those who were not, and of notified cases and patients aged 18–29 years with cervical cancer recorded in the National Cancer Registration Dataset in 2010 (Appendix 1).

Patients were categorised according to whether they had been diagnosed via:

1. Symptomatic presentation: if they had presented to a health professional with one or more symptoms from a checklist of possible symptoms of cervical cancer^5^–^9^,^17^–^19^ (Box 1) and that presentation led to diagnosis (including patients diagnosed via abnormal cytology if the test was done for diagnostic purposes).
2. Abnormality detected on NHS Cervical Screening Programme: if they were diagnosed as a result of a finding during a routine cervical screening test after an invitation on the NHS Cervical Screening Programme.
3. Incidental finding: if they were diagnosed during management of an unrelated condition.

Box 1. **Cervical cancer symptom checklist**

- Bleeding after sex 5–9,17–19
- Bleeding between periods 7,9,17,19
- Change in periods 5
- Bleeding during pregnancy
- Painful sex
- Persistent vaginal discharge 5,6,9,18,19
- Abdominal or pelvic pain or discomfort 7,9,19
- Unusual fatigue

Date of diagnosis was defined as the date the patient said she was told she had cervical cancer.^20^ For patients diagnosed via symptomatic presentation, the ‘trigger’ symptom was identified, defined as the earliest symptom from the checklist in Box 1 that led the patient to present to a health professional and led to diagnosis. For all patients, the symptom that she believed to be the first symptom of her cancer was identified at the time of interview (the ‘first attributed’ symptom) and the earliest symptom from the checklist that the patient reported, but did not attribute to cervical cancer at the time of the interview (the ‘initial’ symptom).

Patient duration of symptoms from onset to first attendance with the trigger symptom was calculated, and provider duration of symptoms from first attendance with trigger symptom to diagnosis, rounding to the nearest month. Delayed presentation was defined as patient duration of symptoms of ≥3 months (a cut-off point that is convention in studies of this nature^21^–^23^), and provider delay as provider duration of symptoms of ≥3 months. Primary care delay was defined as referral ≥6 weeks after first attendance and secondary care delay as diagnosis ≥6 weeks after referral.

For patients who presented via symptomatic presentation and reported provider delay, the general practice records were examined (including free text). Whenever possible, the period in the primary care records which matched the period described at interview, in terms of symptoms and date of attendance (within 3 months of the date reported by the patient), was identified. Records were examined for documented evidence of visualisation of the cervix, tests for genital infection, or cervical cytology. Dates of referral were extracted and used to calculate time from first attendance to referral and from referral to diagnosis.

For patients aged 25–29 years diagnosed via symptomatic presentation, cervical screening history was examined in the national database to find out whether they had been screened and, if so, how long before the presentation, and the result(s).

Odds ratios and 95% confidence intervals (CIs) were calculated to evaluate the association between possible risk factors for delay (Box 2), treated as binary categorical variables, and delay status.

Box 2. **Putative risk factors for patient and provider delay**

**Risk factors for patient delay**

- Nature of the trigger symptom
- Having children
- Having left full-time education aged ≤16 years
- Using hormonal or intrauterine contraception
- Pregnancy
- Not thinking the symptom was serious at the time
- Not knowing what the symptoms of cervical cancer were before diagnosis
- Barriers to symptomatic presentation including:
  - feeling too embarrassed
  - finding it difficult to get an appointment
  - being too busy to make an appointment
  - worrying about not seeing a female doctor
  - worrying about what the GP might find
  - not wanting to waste the GP’s time

**Risk factors for provider delay**

- Nature of the trigger symptom
- Pregnancy
- Using hormonal or intrauterine contraception

The χ^2^ test or Fisher’s exact test (when the expected cell frequency was <5) were used to assess differences between delays and symptom characteristics between age groups and pathways to diagnosis.

All statistical analyses were performed using Stata Statistical Software (Release 12). A *P*-value of <0.05 was considered statistically significant. All statistical tests were two-sided.

## RESULTS

One-hundred and sixteen hospitals identified 333 patients (237 from pathology laboratories and 96 from clinical teams) over an average recruitment period of 8 months (range 1–11 months). One additional patient volunteered to take part after seeing the study on a charity website. Within the study period, 286 (86%) patients were invited; 164 agreed, and 128 (38% of eligible patients identified) were interviewed. Mean time from diagnosis to interview was 4.3 months (range 1.0–8.5 months).

Characteristics of patients identified who were interviewed versus not interviewed were comparable, although interviewed patients were younger and more had adenocarcinoma (Appendix 1). Patients identified were broadly representative of patients identified nationally by the cancer registry for age, stage, histology, and geographical region.

Twenty-two (17%) patients interviewed were aged <25 years. Most were of white ethnic origin, and most had a partner or husband (Table 1). Sixty-two (48%) had children and 32 (25%) had left full-time education at age ≤16 years. Sixty-seven patients (52%) had microinvasive cancer (that is, stage 1a). Ninety-three (73%) had squamous cell carcinoma, 28 (22%) adenocarcinoma, and seven (6%) other histology.

**Table 1.** Demographic and clinical characteristics of interviewed patients

	**Patients aged 18–24 years (*n* = 22), *n* (%)**	**Patients aged 25–29 years (*n* = 106), *n* (%)**	**All patients (*n* = 128), *n* (%)**
**Ethnicity**			
White	22 (100)	99 (93.4)	121 (94.5)

**Age left full-time education, years**			
15–16	8 (36.4)	24 (22.6)	32 (25.0)
17–18	7 (31.8)	38 (35.8)	45 (35.2)
≥19 or still studying	7 (31.8)	44 (41.5)	51 (39.8)

**Had a partner/husband**	21 (95.5)	90 (84.9)	111 (86.7)

**Had children**	13 (59.1)	48 (45.3)	61 (47.7)

**FIGO stage**			
1a	5 (22.7)	62 (58.5)	67 (52.3)
1b	13 (59.1)	40 (37.7)	53 (41.4)
2	2 (9.1)	3 (2.8)	5 (3.9)
3	2 (9.1)	1 (0.9)	3 (2.3)
4	–	–	–

**Histology**			
Squamous	16 (72.7)	77 (72.6)	93 (72.7)
Adenocarcinoma	4 (18.2)	24 (22.6)	28 (21.9)
Other	2 (9.1)	5 (4.7)	7 (5.5)

FIGO = International Federation of Gynecology and Obstetrics.

Of the 22 patients <25 years, one was diagnosed after a cervical lesion was seen during intrauterine device removal and another via screening (just before her 25th birthday); all the others were diagnosed via symptomatic presentation. Of the 106 patients aged 25–29 years, 85 (80%) were diagnosed via an abnormality detected on the NHS Cervical Screening Programme, 20 (19%) were diagnosed via symptomatic presentation, and one was diagnosed after a cervical lesion was seen during insertion of an intrauterine device.

### Patients diagnosed via symptomatic presentation

Of the 40 patients diagnosed via symptomatic presentation, 11 (28%) reported patient delay, and 24 (60%) reported provider delay (Table 2). The most common trigger symptom (33/40) was abnormal vaginal bleeding, with 19 patients describing postcoital bleeding (Table 3).

**Table 2.** Patient and provider delay for patients diagnosed via symptomatic presentation

	**Patients aged 18–24 years (*N* = 20), *n* (%)**	**Patients aged aged 25–29 years (*N* = 20), *n* (%)**	**All patients (*N* = 40), *n* (%)**
**Patient delay**			
Patient duration of symptoms <3 months	12 (60)	17 (85)	29 (73)
Patient duration of symptoms ≥3 months	8 (40)	3 (15)	11 (28)

**Provider delay**			
Provider duration of symptoms <3 months	10 (50)	6 (30)	16 (40)
Provider duration of symptoms ≥3 months	10 (50)	14 (70)	24 (60)

**Table 3.** Frequency of trigger symptoms for patients diagnosed via symptomatic presentation (includes all symptoms, including those of patients who reported more than one symptom, therefore columns do not add up to 40)

	**Patients aged 18–24 years (*N* = 20), *n* (%)**	**Patients aged 25–29 years (*N* = 20), *n* (%)**	**All patients (*N* = 40), *n* (%)**
**Symptom**			
Any bleeding symptom	16 (80.0)	17 (85.0)	33 (82.5)
Bleeding between periods	5 (25.0)	6 (30.0)	11 (27.5)
Bleeding after sex	**10 (50.0)**	**9 (45.0)**	**19 (47.5)**
Bleeding during pregnancy	–	3 (15.0)	3 (7.5)
Change in periods	2 (10.0)	1 (5.0)	3 (7.5)
Painful sex	3 (15.0)	2 (10.0)	5 (12.5)
Vaginal discharge	3 (15.0)	4 (20.0)	7 (17.5)
Abdominal pain	2 (10.0)	3 (15.0)	5 (12.5)

Percentages are calculated based on the total number of patients diagnosed via symptomatic presentation. The most common trigger symptom is in bold.

Twenty-three patients (56%) reported earlier symptoms that had not prompted help-seeking: 10 attributing these symptoms to cancer at the time of interview (six with abnormal vaginal bleeding); and 13 not attributing them to cancer at the time of interview, most commonly vaginal discharge (*n* = 6).

#### Patients aged 18–24 years

Among the 20 patients aged <25 years diagnosed via symptomatic presentation, 16 (80%) had at least stage 1b cancer.

The median duration of symptoms from the trigger symptom to presentation was 2.0 months (interquartile range [IQR] 0.5–4.5 months); 8/20 (40%) had delayed presentation. The median provider duration of symptoms was 3.0 months (IQR 2.0–8.5 months); 10/20 (50%) reported provider delay.

#### Patients aged 25–29 years

Of the 20 patients aged 25–29 years diagnosed via symptomatic presentation, 12 (60%) had at least stage 1b cancer.

Five patients had been diagnosed as a result of abnormal cytology on a diagnostic test after symptomatic presentation. None of these patients had ever attended for cervical screening after a routine invitation.

The median patient duration of symptoms from the trigger symptom was 1.0 month (IQR 0–2.0 months). Fewer patients 3/20 (15%) delayed presentation in comparison with the patients aged <25 years (40%, *P* = 0.16). The median provider duration of symptoms was 6.0 months (IQR 2.5–9.5 months); 14/20 (70%) reported provider delay (Table 2).

Sixteen (80%) of the 20 patients aged 25–29 years with symptomatic presentation consented to access of their screening data. Nine (56%) had attended routine screening within the 3.5 years before diagnosis (that is, were interval cancers), and the rest had never been screened. Of the screened patients, only one had had abnormal cytology but findings had been normal on a subsequent colposcopy 16 months before diagnosis.

#### Risk factors for delayed diagnosis among patients diagnosed via symptomatic presentation

Table 4 shows the frequency of possible risk factors for delays. None of the differences were statistically significant. The largest odds ratio for patient delay, however, was for not knowing what the symptoms of cervical cancer were. For provider delay this was using hormonal or intrauterine contraception.

**Table 4.** Possible risk factors for patient and provider delay for patients diagnosed via symptomatic presentation (*n* = 40)

	**Patients with patient delay (*n* = 11), *n* (%)**	**Patients without patient delay (*n* = 29), *n* (%)**	**Odds ratio (95% CI)**	**Patients with provider delay (*n* = 24), *n* (%)**	**Patients without provider delay (*n* = 16), *n* (%)**	**Odds ratio (95% CI)**
**Symptom**						
Nature of trigger symptom (*n* = 40)						
Any bleeding symptom	9 (82)	24 (83)	0.9 (0.1 to 11.5)	20 (83)	13 (81)	1.2 (0.1 to 8.1)
Bleeding between periods	2 (18)	9 (31)	0.5 (0.0 to 3.2)	6 (25)	5 (31)	0.7 (0.1 to 3.9)
Bleeding after sex	6 (55)	13 (45)	1.5 (0.3 to 7.6)	11 (46)	8 (50)	0.8 (0.2 to 3.6)
Painful sex	1 (9)	4 (14)	0.6 (0.0 to 7.5)	3 (13)	2 (13)	1.0 (0.1 to 13.4)
Vaginal discharge	3 (27)	4 (14)	2.3 (0.3 to 17.0)	3 (13)	4 (25)	0.4 (0.1 to 3.1)
Did not think the trigger symptom was serious at the time	10 (91)	23 (79)	2.6 (0.3 to 131.8)	–	–	
**Demographic factors**						
Had children	7 (64)	12 (41)	2.5 (0.5 to 14.0)	–	–	
Left school aged ≤16 years	3 (27)	5 (17)	1.8 (0.2 to 11.8)	–	–	

**Other gynaecological factors**						
Using hormonal contraception or intrauterine device ^a^	8 (89)	17 (81)	1.9 (0.1 to 104.1)	15 (88)	10 (77)	2.3 (0.2 to 30.7)
Pregnant at first attendance (*n* = 3) ^b^	0	3 (10)	–	3 (13)	0	–

**Reported knowledge of cervical cancer symptoms before diagnosis**						
Did not know what the symptoms of cervical cancer were	10 (91)	20 (69)	4.5 (0.5 to 216.6)	–	–	

**Barriers to symptomatic presentation^c^**						
Feeling too embarrassed	6 (55)	8 (29)	3.0 (0.6 to 16.2)	–	–	
Worrying about not seeing a female doctor	5 (45)	8 (29)	2.1 (0.4 to 11.0)	–	–	
Finding it difficult to get an appointment	6 (55)	10 (37)	2.0 (0.4 to 10.7)	–	–	
Not wanting to waste the GP’s time	5 (45)	11 (39)	1.3 (0.2 to 6.5)	–	–	
Being too busy to make an appointment	6 (55)	14 (48)	1.3 (0.3 to 6.6)	–	–	
Worrying about what the GP might find	2 (18)	5 (18)	1.0 (0.1 to 7.8)	–	–	

a Percentages calculated excluding pregnant patients from the denominator.

b Percentages calculated using the number of pregnant patients as the denominator.

c Missing values for some patients, therefore percentages were calculated using the number of patients who answered each question as the denominator.

### Explaining provider delays among patients who presented symptomatically

Primary care records were received for 21/24 patients who reported a provider delay after symptomatic presentation. For three of these, however, the first attendance had been at a sexual health clinic, meaning there were no records of the relevant consultations. For the remaining 18, a consultation could be identified in the GP notes matching the first attendance reported by the patient (in terms of similar symptoms and date) for 10 (56%) patients. For six of these patients, the delay occurred in primary care. For two of these, there was no record of visualising the cervix. Both had presented with intermenstrual bleeding (one also had postcoital bleeding) and both had tested negative for genital infection. In both of these patient’s records, the records documented advice to return if symptoms continued; however, for both, the subsequent attendance was 5–6 months later.

For the other four patients with primary care delay, visualisation of the cervix was recorded: normal in two, cervical polyp for one, and ‘cervical bleeding on contact’ for one. The notes recorded tests for genital infections in all four patients, of whom three had positive results; one chlamydia and two bacterial vaginosis. For three of these patients, 2–6 months elapsed between first presentation and the next attendance. Advice to re-attend was documented in only one of the patient’s GP notes. The remaining patient was referred routinely to gynaecology after 3 months of re-presenting with heavy intermenstrual bleeding.

For the remaining four patients, there was secondary care delay. In three of these, no malignancy was found on the initial biopsy in secondary care. The remaining patient waited 8–10 weeks to be seen in gynaecology, then colposcopy.

### Patients who were pregnant

Three patients (8%) diagnosed via symptomatic presentation were pregnant at the time of first presentation. All three had reported bleeding during their pregnancy, presented straight away, and were managed in secondary care. Two of these patients had a colposcopy while pregnant, but no malignancy was identified on biopsy. All three patients continued to have symptoms throughout pregnancy and after giving birth, and were eventually diagnosed 9–10 months after presenting with their trigger symptom (stages 1b1, 2b, and 3b).

### Patients diagnosed via an abnormality detected on NHS Cervical Screening Programme

Most patients diagnosed via routine cervical screening had microinvasive cancer (55/86, 64%). Most (72/86; 84%) reported symptoms that started before the diagnostic screening test, most commonly vaginal discharge (*n* = 39), postcoital bleeding (*n* = 38), and intermenstrual bleeding (*n* = 23).

More patients diagnosed via screening reported longstanding symptoms (>2 years) than those diagnosed via symptomatic presentation (for example, postcoital bleeding 42% [16/38] versus 17% [5/30]; *P* = 0.02, intermenstrual bleeding 43% [10/23] versus 18% [4/22]; *P* = 0.07). Also, abnormal vaginal bleeding was less severe in screen-detected patients with fewer reporting flooding or clots (19% [10/53] versus 44% [17/39], *P* = 0.01).

About half of the patients diagnosed via screening who reported symptoms (35/72; 49%) reported that they had presented with these to a health professional. Their most common symptoms were abnormal vaginal bleeding (16/35, 46%) (mainly postcoital bleeding [10/35, 29%]) and dyspareunia (10/35, 29%). Median duration of symptoms from first symptom to diagnosis was 29.6 months (IQR 15.4–87.1 months).

The remaining 37 patients who reported symptoms said they had not sought help for their symptoms. Their most common symptoms were vaginal discharge (16/37, 43%), postcoital bleeding (13/37, 35%) and abdominal pain and fatigue (both 12/37, 32%). Median duration of symptoms from first symptom to diagnosis was 17.8 months (IQR 7.7–73.4 months).

## DISCUSSION

### Summary

Among 128 young patients diagnosed with cervical cancer aged 18–29 years, 40 were diagnosed via symptomatic presentation. These patients were more likely to have more advanced cancers than patients who were diagnosed via screening, although very few patients had stage 2 or above. Of the patients eligible for screening, one-fifth were diagnosed via symptomatic presentation.

Most patients presenting symptomatically had abnormal vaginal bleeding, most commonly postcoital bleeding. One-quarter of these delayed presentation for ≥3 months after onset of symptoms: this was more common in those aged <25 years. Over half reported gynaecological symptoms some time before the symptom that caused them to present: this may have been an opportunity for earlier diagnosis. Vaginal discharge was the most common of these.

Provider delay was reported frequently. No conclusive evidence was found about risk factors for provider delay, although not all patients with provider delay had had their cervix visualised in primary care and many had co-existing genital infection. There was also some evidence that some delay in primary care may be because of patients not re-attending after first presentation promptly despite persistent symptoms.

### Strengths and limitations

To the authors’ knowledge, this study has provided the most detailed information to date on the nature and duration of symptoms for young females with cervical cancer. The methods and reporting are consistent with the Aarhus statement on studies of early cancer diagnosis.^20^ In the present study, a high identification rate was achieved and the population was broadly representative of patients diagnosed with cervical cancer in England over a similar period. Another key strength was the high quality of the data collected. By using an interview-based measure, which was developed and tested in young patients with cervical cancer,^15^ it was possible to record complicated data systematically.

The possibility of recall error cannot be ruled out. This was minimised using calendar anchoring and collaborative completion of a timeline detailing the events that led to diagnosis.^15^ In addition, most patients were interviewed within 6 months of diagnosis.

Identification of risk factors for delays in cervical cancer diagnosis was limited by small study numbers, which is inevitable with rare diseases. A study with sufficient power to find statistically significant risk factors for delay in presentation would have to recruit patients for years.

### Comparison with existing literature

The present findings are consistent with research showing that cervical cancer symptom awareness among young British females is low.^24^ An online YouGov survey of 2726 females in the UK found that 20% of 18–24-year-olds said that they would not seek help for bleeding during sex (M Durrant, personal communication, August 2013). A systematic review that examined the risk factors for delayed presentation in cancer found that non-recognition of the severity of symptoms, symptom type (for example, vague), infrequent care-seeking, and fear were the predominant risk factors for delayed presentation for gynaecological cancers.^25^

### Implications for research and practice

The study confirms the findings of case reports suggesting that patients <25 years often delay presentation with cervical cancer and have more advanced stage. This supports the idea that earlier presentation could improve outcomes in this group.

Although this study was small, it provides preliminary data that could be used to inform interventions to promote earlier presentation in this group by ensuring that they set out the possible symptoms of cervical cancer clearly (framing these in particular for young females of low education, who are at higher risk of cervical cancer in any case),^26^ perhaps emphasising that other priorities, embarrassment, and worry about wasting the doctor’s time should not deter them from presenting, and that they will be able to see a female doctor if they wish.

The challenge is that gynaecological symptoms in young females are very common, and promoting early presentation for these symptoms could lead to a large increase in attendances in primary care and unnecessary anxiety in females who do not have cancer (or cervical abnormalities). For females aged 15–29 years, at least 1.6% presented to primary care with intermenstrual bleeding, 0.5% with postcoital bleeding, and 1.3% with vaginal discharge in any 1-year period.^11^

Although most screen-detected patients reported symptoms, a high proportion of these were longstanding yet most had early stage cancer. This implies that some of their symptoms were unlikely to have been related to their cancer.

The present data suggest that pregnancy at the time of presentation may be a risk factor for provider delay; however, these patients appear to be difficult to diagnose given that even at colposcopy (including biopsy), malignancy was not easily identified.

The data from primary care records were too sparse to be able to draw any firm conclusions about the quality of management of gynaecological symptoms in primary care, although patients may not be having their cervix visualised when they present with abnormal vaginal bleeding, despite the UK guidance on this.^17^ Furthermore, patients may delay re-attending after the first presentation, and re-attendance should be strongly advised if their symptoms persist.

## Appendix

**Appendix 1.** Characteristics of patients aged 18–29 years diagnosed with cervical cancer identified over the study period who responded, who were interviewed versus not interviewed and patients identified over the study period versus patients aged 18–29 years diagnosed with cervical cancer in England in 2010

	**Patients interviewed (*N* = 128), *n* (%)**	**Patients not interviewed (*N* = 206), *n* (%)**	**Patients diagnosed in 2010 ^a^ (*N* = 352), *n* (%)**	**Patients identified over study period (*N* = 334), *n* (%)**
**Age at diagnosis, years**				
18–24	22 (17)	10 (5)	45 (13)	32 (10)
25–29	106 (83)	196 (95)	307 (87)	302 (90)

**FIGO stage ^b^**				
1a	67 (52)	127 (62)	267 (88)	303 (93)
1b	52 (41)	56 (27)		
2	6 (5)	12 (6)	22 (7)	17 (5)
3	3 (2)	1 (0.5)	6 (2)	4 (1)
4	0	2 (1)	7 (2)	7 (2)
Missing	0	8 (3)	50	8

**Histology**				
Squamous cell carcinoma	93 (73)	170 (83)	276 (78)	263 (79)
Adenocarcinoma	28 (22)	28 (14)	47 (13)	56 (17)
Other	7 (5)	6 (3)	29 (8)	13 (4)
Missing	–	2 (1)	–	2 (1)

**Strategic Health Authority**				
North East	19 (9)	7 (5)	28 (8)	26 (8)
North West	35 (17)	17 (13)	64 (18)	52 (16)
Yorkshire & Humber	38 (18)	24 (19)	49 (14)	62 (19)
East Midlands	19 (9)	14 (11)	34 (10)	33 (10)
West Midlands	27 (13)	15 (12)	38 (11)	42 (13)
East of England	21 (10)	11 (9)	33 (9)	32 (10)
London	14 (7)	15 (12)	27 (8)	26 (8)
South East Coast	5 (2)	4 (2)	17 (5)	20 (6)
South Central	14 (7)	7 (5)	21 (6)	21 (6)
South West	14 (7)	6 (5)	41 (12)	20 (6)

Some percentages do not add up to 100% due to rounding.

a Data supplied by Trent Cancer Registry from the 2010 National Cancer Data Repository.

b For patients diagnosed in England in 2010 and patients identified over the study period, percentages were calculated using the number of patients with known FIGO stage as the denominator because of the high proportion of patients with missing stage. FIGO = International Federation of Gynecology and Obstetrics.

## References

[1] Sasieni P, Adams J, Cuzick J (2003). Benefit of cervical screening at different ages: evidence from the UK audit of screening histories. Br J Cancer.

[2] Reynolds E (2012). Woman, 23, died of cervical cancer ‘because doctors said she was too young for a smear test’. Mail Online.

[3] (2013). Jo’s Trust. Case studies: Stacey’s story.

[4] Advisory Committee On Cervical Screening (ACCS) Extraordinary Meeting to re-examine current policy on cervical screening for women aged 20–24 years taking account of any new evidence and to make recommendations to the National Cancer Director and Ministers.

[5] Pretorius R, Semrad N, Watring W, Fotheringham N (1991). Presentation of cervical cancer. Gynecol Oncol.

[6] Shapley M, Jordan J, Croft PR (2006). A systematic review of postcoital bleeding and risk of cervical cancer. Br J Gen Pract.

[7] van Schalkwyk SL, Maree JE, Wright SC (2008). Cervical cancer: the route from signs and symptoms to treatment in South Africa. Reprod Health Matters.

[8] Viikki M, Pukkala E, Hakama M (1998). Bleeding symptoms and subsequent risk of gynecological and other cancers. Acta Obstet Gynecol Scand.

[9] Yu CK, Chiu C, McCormack M, Olaitan A (2005). Delayed diagnosis of cervical cancer in young women. J Obstet Gynaecol.

[10] O’Dowd TC, Parker S, Kelly A (1996). Women’s experiences of general practitioner management of their vaginal symptoms. Br J Gen Pract.

[11] Stapley S, Hamilton W (2011). Gynaecological symptoms reported by young women: examining the potential for earlier diagnosis of cervical cancer. Fam Pract.

[12] Office for National Statistics (2013). Cancer Registration Statistics, England, 2011.

[13] Office for National Statistics (2012). Cancer Statistics Registrations, England 2010.

[14] NHS Information Centre for Health and Social Care Cervical Screening Programme — England, 2008–2009.

[15] Lim AW, Forbes LJ, Rosenthal AN (2013). Measuring the nature and duration of symptoms of cervical cancer in young women: developing an interview-based approach. BMC Womens Health.

[16] Andersen RS, Vedsted P, Olesen F (2009). Patient delay in cancer studies: a discussion of methods and measures. BMC Health Serv Res.

[17] Department of Health Clinical practice guidelines for the assessment of young women aged 20–24 with abnormal vaginal bleeding.

[18] Department of Health (2010). Cervical cancer key messages 2010.

[19] Scottish Intercollegiate Guidelines Network (SIGN) Management of cervical cancer 2008; (Guideline No. 99).

[20] Weller D, Vedsted P, Rubin G (2012). The Aarhus statement: improving design and reporting of studies on early cancer diagnosis. Br J Cancer.

[21] Ramirez AJ, Westcombe AM, Burgess CC (1999). Factors predicting delayed presentation of symptomatic breast cancer: a systematic review. Lancet.

[22] Richards MA, Westcombe AM, Love SB (1999). Influence of delay on survival in patients with breast cancer: a systematic review. Lancet.

[23] Pack GT, Gallo JS (1938). The culpability for delay in treatment of cancer. Am J Cancer.

[24] Low EL, Simon AE, Lyons J (2012). What do British women know about cervical cancer symptoms and risk factors?. Eur J Cancer.

[25] Macleod U, Mitchell ED, Burgess C (2009). Risk factors for delayed presentation and referral of symptomatic cancer: evidence for common cancers. Br J Cancer.

[26] Brown J, Harding S, Bethune A, Rosato M (1997). Incidence of health of the nation cancers by social class. Popul Trends.

